# A Targeted Quantitative Proteomic Method Revealed a Substantial Reprogramming of Kinome during Melanoma Metastasis

**DOI:** 10.1038/s41598-020-59572-5

**Published:** 2020-02-12

**Authors:** Weili Miao, Lin Li, Xiaochuan Liu, Tianyu F. Qi, Lei Guo, Ming Huang, Yinsheng Wang

**Affiliations:** 1 Department of Chemistry, University of California, Riverside, CA 92521-0403 USA; 2 Environmental Toxicology Graduate Program, University of California, Riverside, CA 92521-0403 USA

**Keywords:** Mass spectrometry, Proteomics

## Abstract

Kinases are involved in numerous critical cell signaling processes, and dysregulation in kinase signaling is implicated in many types of human cancers. In this study, we applied a parallel-reaction monitoring (PRM)-based targeted proteomic method to assess kinome reprogramming during melanoma metastasis in three pairs of matched primary/metastatic human melanoma cell lines. Around 300 kinases were detected in each pair of cell lines, and the results showed that Janus kinase 3 (JAK3) was with reduced expression in the metastatic lines of all three pairs of melanoma cells. Interrogation of The Cancer Genome Atlas (TCGA) data showed that reduced expression of JAK3 is correlated with poorer prognosis in melanoma patients. Additionally, metastatic human melanoma cells/tissues exhibited diminished levels of *JAK3* mRNA relative to primary melanoma cells/tissues. Moreover, JAK3 suppresses the migration and invasion of cultured melanoma cells by modulating the activities of matrix metalloproteinases 2 and 9 (MMP-2 and MMP-9). In summary, our targeted kinome profiling method provided by far the most comprehensive dataset for kinome reprogramming associated with melanoma progression, which builds a solid foundation for examining the functions of other kinases in melanoma metastasis. Moreover, our results reveal a role of JAK3 as a potential suppressor for melanoma metastasis.

## Introduction

Kinases constitute crucial elements in cell signaling, and in regulation of cell proliferation and metabolism^1,2^. Aberrant expression and/or activation of kinases have been observed in cancer and many other human diseases^3^. Melanoma is a common type of cancer, and its metastasis leads to the majority of mortality in melanoma patients^4^. Aberrant kinase activation is associated with melanoma. For instance, BRAF V600E and V600K mutations, which lead to constitutive activation of the kinase, are among the most frequent genetic alterations found in melanoma patients^5^. Dabrafenib and vemurafenib, which bind to BRAF and inhibit its kinase activity, are Food and Drug Administration (FDA)-approved drugs for the treatment of metastatic melanoma^6^. However, patients treated with these BRAF inhibitors relapse at 6–8 months after therapy^7^. Immunotherapy relying on checkpoint blockade can allow for relapse-free remission of melanoma; the response rate, nevertheless, is low^8^. Hence, it’s important to develop new and effective therapeutic approaches for melanoma treatment. We reason that the identification of those kinases that promote or suppress melanoma metastasis will improve our understanding about the etiology of melanoma progression and may reveal novel targets for its therapeutic intervention.

Several methods have been developed for the quantification of kinase proteins at the entire proteome level. For example, isotope-coded ATP-affinity probes were shown to be effective in the covalent labeling and enrichment of kinase proteins or their component peptides for subsequent LC-MS/MS analysis^9–11^. Both the protein expression level of a kinase and its ATP-binding affinity can affect the extent of kinase labeling by the ATP affinity probe, where the ATP binding affinity of a kinase is sometimes modulated by its activity^12^. Additionally, affinity resin immobilized with various kinase inhibitors was utilized for kinase protein enrichment, where the enrichment efficiency may also be impacted by kinase activity^13–15^. Recently, we developed a parallel-reaction monitoring (PRM)-based targeted proteomic method for assessing specifically the kinase protein expression at the entire proteome scale^12^. In this regard, we established a Skyline LC-PRM library for kinome analysis based on the shotgun proteomic data collected by analyzing tryptic digestion mixtures from multiple human cell lines^12^. The library encompasses 1050 tryptic peptides representing 478 kinase proteins, and 395 of them are protein kinases^12,16–18^.

In this work, we applied the recently established PRM-based targeted proteomic method^12^, in combination with stable isotope labeling by amino acids in cell culture (SILAC), to examine the differential protein expression of kinases in 3 pairs of matched primary/metastatic melanoma cells. We were able to quantify the relative expression levels of approximately 300 unique kinase proteins in each pair of cell lines. Among them, JAK3, MAP4K4 and CCNH were consistently down-regulated in the metastatic lines of all three pairs of melanoma cells. Analysis of publicly available data also showed that elevated JAK3 expression is correlated with improved survival of melanoma patients. We further demonstrated that JAK3 suppresses the migratory and invasive abilities of melanoma cells *in vitro*, which involves diminished enzymatic activities of secreted matrix metalloproteinases.

## Results

### Quantitative assessment of the differential expression of kinase proteins in primary and metastatic human melanoma cells

**Figure 1:**
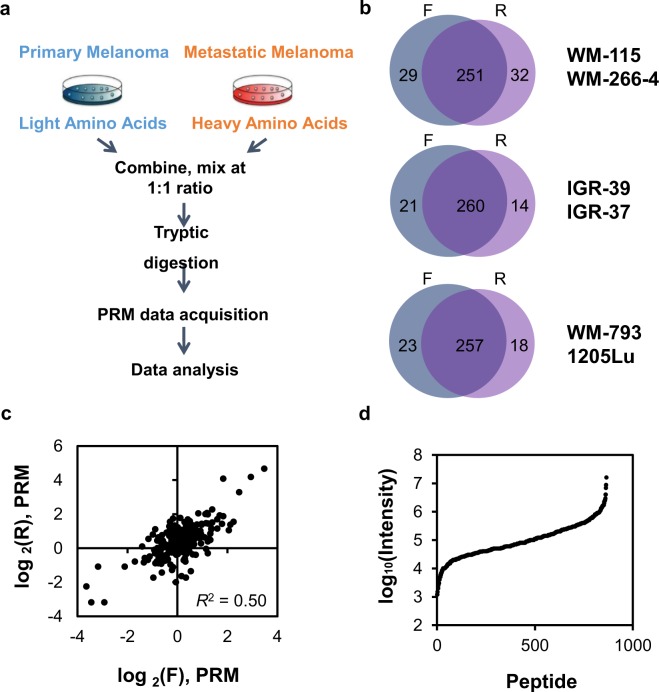
LC-PRM analysis, in conjunction with metabolic labeling using SILAC, for interrogating the differential expression of kinase proteins in paired primary/metastatic melanoma cells. (**a**) A schematic diagram showing the forward SILAC-based targeted proteomics workflow for human kinome analysis in paired primary/metastatic human melanoma cells. (**b**) Venn diagrams showing the overlap between the numbers of kinases quantified from forward and reverse SILAC labeling experiments for the three pairs of primary/metastatic melanoma cells. (**c**) Correlation between the log_2_(Ratio) in kinase protein expression in WM-266-4 over WM-115 cells obtained from forward and reverse SILAC labeling experiments. (**d**) Distribution of signal intensities for the quantified kinase peptides. The detailed quantification data, including the list of peptides employed for LC-PRM analyses, are shown in Table S1.

Our primary objective was to explore the potential roles of kinases in melanoma metastasis. Toward this end, we applied a PRM-based targeted proteomic method^12^, in combination with SILAC^19^, to examine the differences in kinase protein expression in 3 pairs of primary/metastatic melanoma cells (i.e. IGR-39/IGR-37, WM-115/WM-266-4, and WM-793/1205Lu) (Fig. 1a). In this vein, WM-115 and WM-266-4 cells were derived from the primary and skin metastatic sites of the same melanoma patient, respectively^20^, and IGR-39 and IGR-37 cells were initiated respectively from the primary and groin metastatic sites of another melanoma patient^21^. On the other hand, 1205Lu cells were originated from a lung metastasis in an immune-deficient mouse through tail-vein injection of WM-793 human melanoma cells, which were initiated from a superficial spreading melanoma^22^.

The proteomic results led to the quantification of approximately 300 unique kinases in each pair of melanoma cells, including 237–252 protein kinases, along with many lipid, carbohydrate and nucleotide kinases (Figs. S1–S3, Tables 1 and S1). All 4–6 PRM transitions used for the quantification of every kinase peptide displayed the same retention time and exhibited a dot product (dotp) value of >0.7 (Fig. S4)^23^. In addition, over 90% of the quantified kinase peptides showed consistent trends in forward and reverse SILAC labeling experiments (Fig. 1b,c, Table S1, and representative results for AK1 are shown in Fig. S5b). Along this line, owing to the complex matrices for the samples employed for the LC-PRM analysis (i.e. the tryptic digestion mixtures of whole-cell protein lysates), a somewhat modest *R*^2^ value (0.50) was found for the correlation between the ratios obtained from forward and reverse SILAC labeling experiments (Fig. 1c). Based on the range of signal intensities detected for the kinase peptides, we estimated that the PRM method exhibits a dynamic range of 4 orders of magnitude (Fig. 1d).

**Table 1:** The numbers of protein, lipid, nucleotide, and carbohydrate kinases quantified in the three pairs of primary/metastatic melanoma cells.

	WM-115/WM-266-4	IGR-39/IGR-37	WM-793/1205Lu
Protein kinases	252	237	242
Lipid kinases	10	11	10
Nucleotide kinases	27	27	28
Carbohydrate kinases	12	13	12
Other kinases	5	6	6

**Figure 2:**
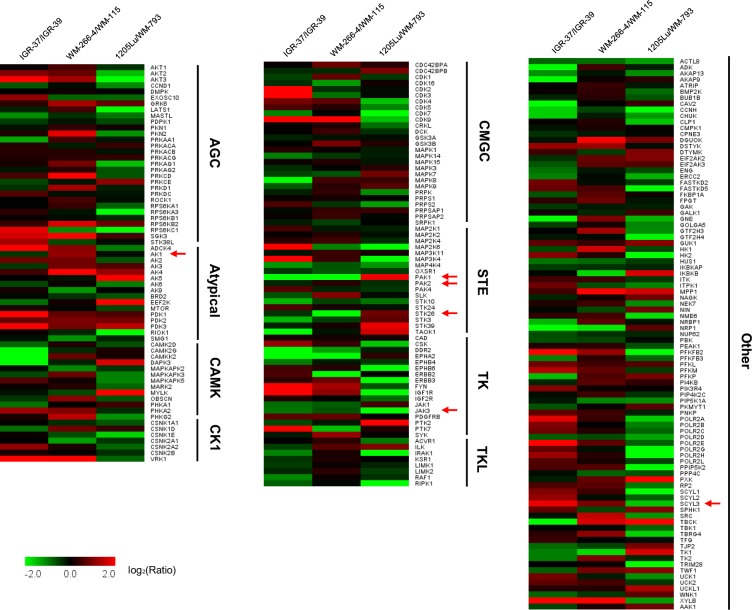
A heatmap showing the differential expression of kinase proteins in 3 pairs of matched primary/metastatic melanoma cell lines. The data represent the mean values of the results obtained from four replicates (two forward and two reverse SILAC labeling experiments) for the WM-115/WM-266-4 pair and two replicates each (one forward and one reverse SILAC labeling experiments) for the IGR-39/IGR-37 and WM-793/1205Lu pairs (see Table S1 for ratios obtained from individual biological replicates). Only those kinases that were commonly quantified in all three pairs of melanoma cells were plotted. Red and green boxes represent the up- and down-regulated kinases in metastatic over primary melanoma cells, respectively. Arrows indicate those kinases whose relative levels of expression in the 3 matched pairs of melanoma cells were validated by Western blot analyses (see Fig. 3).

A comparison of the kinome quantification results revealed that the IGR-39/IGR-37and WM-115/WM-266-4 pairs of melanoma cells displayed many similar attributes in metastasis-associated alterations in expression levels of kinases. However, the results for these two pairs differed substantially from those obtained for the WM-793/1205Lu pair (Fig. 2 and Table S1). This might be attributed to the facts that melanoma is a highly heterogeneous disease^24^ and that the metastatic lines from the first two pairs were derived from metastasis in melanoma patients, whereas that of the last pair was from experimental metastasis in a mouse^22^.

**Figure 3:**
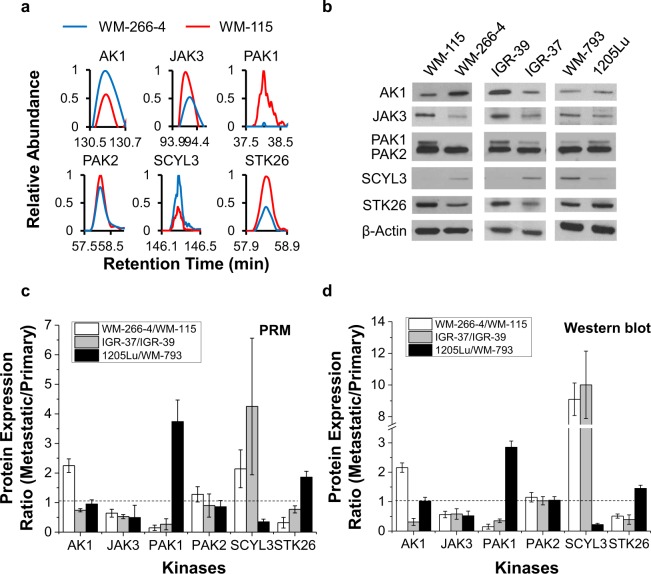
Validation of quantification results for kinases obtained from the PRM-based targeted proteomic method. (**a**) Representative PRM traces for peptides from targeted kinases. Listed are the PRM traces of IGQPTLLLYVDAGPETMTQR from AK1 (y_5_, y_6_, y_7_, y_8_, y_9_, y_10_), SCSPSAEFLR from JAK3 (y_3_, y_5_, y_6_, y_7_, y_8_), NTSTMIGAGSK from PAK1 (y_5_, y_6_, y_7_, y_8_, y_9_), MTDEEIMEK from PAK2 (y_3_, y_4_, y_5_, y_6_, y_7_, y_8_), VILPQVLLGLR from SCYL3 (y_4_, y_5_, y_6_, y_7_, y_8_, y_9_) and SIAVAEAACPGITDK from STK26 (y_3_, y_6_, y_7_, y_8_, y_9_, y_11_). The y ions listed in the parentheses were the fragment ions observed in the MS/MS of the corresponding peptides that were chosen for peptide quantification. (**b**) Western blot for the validation of the expression levels of representative kinases in paired primary/metastatic melanoma cells. (**c**) Quantitative comparisons of ratios of kinase protein expression obtained from PRM (**c**) and Western blot (**d**) analyses. The data represent the mean ± S. D. of the quantification results (n = 3). The uncropped images for Western blot are provided in supporting information file.

We also validated the protein expression levels for six quantified kinases (AK1, JAK3, PAK1, PAK2, SCYL3 and STK26) by Western analysis and the results are all in agreement with what we obtained from PRM measurement (Fig. 3), supporting that the LC-PRM method, in conjunction with SILAC labeling, afforded robust quantification of kinase proteins. In this vein, it’s worth noting that the PRM-based LC-MS/MS quantification method relies on unique peptide sequences of kinases; hence, the method can distinguish different kinase isoforms. For instance, the method allowed for the independent quantifications of PAK1 and PAK2 (Fig. 3c). The PAK antibody that we used for Western blot analysis can recognize all three isoforms of PAK (i.e. PAK1, PAK2, and PAK3). While the peptides derived from PAK3 were below the detection limit of the PRM method, the peak intensity observed for the PAK2 peptide is approximately 10-fold higher than that for the PAK1 peptide, which is consistent with the Western blot results. These observations, in conjunction with the similar molecular weights of PAK1 and PAK3, allow us to assign the higher-molecular weight band observed in Western blot exclusively to PAK1.

We further performed KEGG pathway analysis^25^ on the basis of those kinases that are up-regulated in the metastatic WM-266-4, IGR-37 and 1205Lu cells over the corresponding primary melanoma cells, and we found that many pathways, including those of MAPK signaling, cancer, focal adhesion and purine metabolism, were up-regulated (Fig. S6). Additionally, pathways in melanoma is up-regulated based on those kinases that are commonly up-regulated in the metastatic WM-266-4 and IGR-37 cells over the corresponding primary melanoma cells (Fig. S6).

### Potential roles of JAK3 in melanoma progression

**Figure 4:**
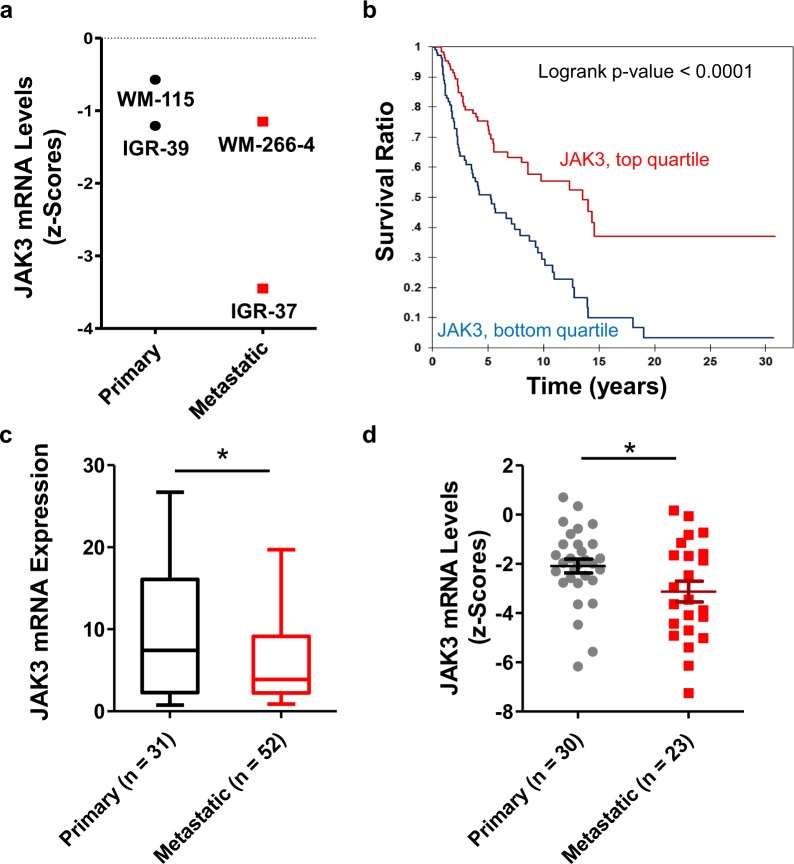
Potential roles of JAK3 in melanoma progression. (**a**) The mRNA expression levels of *JAK3* in WM-115, WM-266-4, IGR-39, and IGR-37 cell lines from CCLE database. (**b**) Kaplan-Meier plot showing the relationship between the mRNA expression levels of *JAK3* gene and the overall survival of melanoma patients. The data were retrieved from TCGA database and analyzed using OncoLnc (http://www.oncolnc.org/). (**c**,**d**) The mRNA expression levels of *JAK3* are down-regulated in metastatic melanoma tissues and cells relative to primary melanoma tissues and cells. Primary and metastatic melanoma patients in the GSE8401 dataset (**c**) and cells in the CCLE database (**d**) were employed for analysis. The displayed boxes contain the interquartile levels or z-scores of mRNA of *JAK3* gene obtained from patients and cells. Shown by the whiskers extending outside of the box are the maximum and minimum mRNA level or z-scores of JAK3 expression from patients and cells.

To identify potential drivers and suppressors for melanoma metastasis, we compared the 130 perturbed kinases obtained from the PRM results of WM-115/WM-266-4 pair of cells with the TCGA data collected for more than 400 melanoma patients^26^. We found that the mRNA expression levels of 19 differentially expressed kinases are correlated with the overall survival of melanoma patients (Table S2, Logrank p-value < 0.05). Among these kinases, 6 (RPS6KB2, DSTYK, TBRG4, CDK4, POLR2E, FASTKD5) and 4 (STK26, PAK1, EPHB2 and JAK3) were consistently up- or down-regulated, respectively, in the metastatic lines of at least two pairs of melanoma cells (Figs. 4b and S7). For instance, JAK3 displayed consistently lower levels of expression in all three metastatic lines of the paired melanoma cells (Fig. 3c,d). The same trend held for the mRNA expression levels (Fig. 4a).

JAK3, a tyrosine kinase involved in signal transduction by receptors^27^, was previously shown to assume important roles in multiple types of cancer. For instance, JAK3 was demonstrated to promote breast cancer metastasis^28^. In addition, JAK3 exhibits high level of expression in leukemia (Fig. S8) and it was found to be frequently mutated in T-cell acute lymphoblastic leukemia^29^. Furthermore, JAK3 was shown to function together with STAT3 to control cell migration, invasion and apoptosis^30^. The roles of JAK3 in melanoma progression, however, remain unclear. To address this, we first investigated whether *JAK3* gene is expressed at different levels in metastatic and primary melanoma tissues by analyzing the data retrieved from the Gene Expression Omnibus (GEO) and CCLE datasets. In particular, an analysis of the GSE8401 dataset, which included 83 melanoma patients (31 primary and 52 metastatic)^31^, revealed a significant down-regulation of *JAK3* gene in the metastatic relative to primary melanoma tissues (Fig. 4c). Analysis of the CCLE dataset also unveiled the diminished mRNA level of *JAK3* gene in metastatic melanoma cell lines over primary melanoma lines (Figs. 4d and S8). Thus, our quantitative proteomic results, combined with TCGA data and other datasets, suggest that JAK3 may suppress melanoma metastasis.

### JAK3 suppresses the invasion of cultured melanoma cells by regulating the activities of secreted metalloproteinases (MMPs)

**Figure 5:**
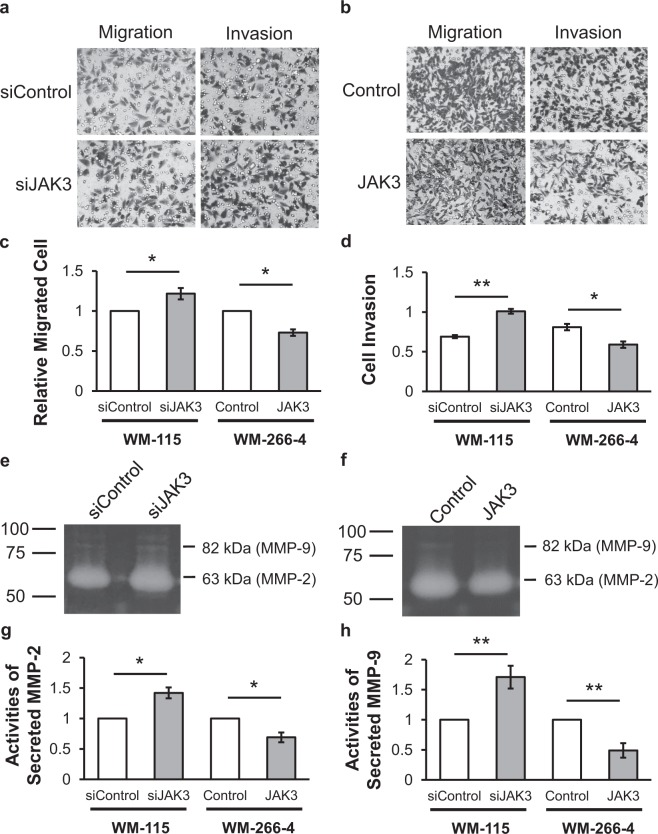
JAK3 modulates the migratory and invasive capacities of cultured melanoma cells through regulating the enzymatic activities of MMP-2 and MMP-9. (**a**) The migratory and invasive abilities of WM-115 primary melanoma cells upon siRNA-mediated knock-down of *JAK3* gene. (**b**) The migratory and invasive abilities of WM-266-4 metastatic melanoma cells upon ectopic overexpression of *JAK3* gene. (**c**,**d**) Quantification results for the migratory (**c**) and invasive (**d**) capacities of WM-115 cells upon knock-down of *JAK3* gene and of WM-266-4 cells upon overexpression of *JAK3* gene. (**e**,**f**) Gelatin zymography assays showing the changes in activities of secreted MMP-2 and MMP-9 after siRNA-mediated knock-down of *JAK3* gene in WM-115 cells (**e**) and upon ectopic overexpression of *JAK3* gene in WM-266-4 cells (**f**). Shown on the left are the locations of molecular weight size markers (in kDa). (**g**,**h**) Quantification results for the activities of secreted MMP-2 (**g**) and MMP-9 (**h**) in WM-115 upon knock-down of *JAK3* and in WM-266-4 cells upon ectopic overexpression of *JAK3*. The data represent the mean ± S. D. of the quantification results (n = 3). The *p* values were calculated based on unpaired, two-tailed Student’s *t*-test: #, *p* ≥ 0.05; *0.01 ≤ *p* < 0.05; **0.001 ≤ *p* < 0.01; ***p < 0.001.

We next studied the potential function of JAK3 in melanoma metastasis by asking how the capacities in migration and invasion of cultured melanoma cells are influenced by the expression levels of kinase proteins^32^. Our results from transwell migration and invasion assay showed that the WM-115 primary melanoma cells exhibited a modest, yet statistically significant increase in migratory capacity, and a marked elevation in invasive ability upon siRNA-mediated knock-down of JAK3 (Figs. 5a,c,d and S9). Reciprocal experiment showed that the overexpression of JAK3 in WM-266-4 metastatic melanoma cells suppressed their motility and invasion (Figs. 5b–d and S9).

Given that MMP-2 and MMP-9 play important roles in degrading extracellular matrix proteins and promoting cancer metastasis^33^, we next investigated whether the enzymatic activities of MMP-2 &-9 could be modulated by JAK3 expression levels. We found, from gelatin zymography assay^34^, that the activities of secreted MMP-2 MMP-9 were heightened in WM-115 primary melanoma cells upon siRNA-mediated knock-down of JAK3 (Figs. 5e,g,h and S9). Reciprocally, ectopic overexpression of JAK3 in WM-266-4 metastatic melanoma cells led to diminished enzymatic activities of secreted MMP-2 &9 (Figs. 5f–h and S9). Together, the above results suggest that JAK3 impedes the invasion of melanoma cells *in vitro*, at least in part, by regulating the activities of secreted MMP-2 and MMP-9.

The epithelial-to-mesenchymal transition (EMT) is often accompanied with invasion of cancer cells, particularly those of epithelial origin^35^, and JAK3 was shown to inhibit EMT by strengthening the adherens junctions^36^. Hence, we also examined the levels of expression of N-cadherin, an EMT marker, and our result showed that the N-cadherin protein was expressed at a higher level in WM-115 primary melanoma cells than WM-266-4 metastatic melanoma cells (Fig. S10). Furthermore, siRNA-mediated knockdown of JAK3 resulted in diminished expression of N-cadherin (Fig. S10). Thus, EMT does not appear to contribute to elevated invasive capacity of the WM-266-4 melanoma cells, and the JAK3-mediated suppression of invasive capacity of melanoma cells is not attributed to EMT inhibition. This is in keeping with the previous finding that melanoma cells are not of epithelial origin^37^.

## Discussion

Kinase- and phosphatase-mediated reversible phosphorylation of proteins and small molecules represents one of the most crucial and best characterized cell signaling pathways^1,2^. Thus, high-throughput kinome profiling constitutes a powerful approach for the systematic interrogation of kinase-modulated molecular events forged by extracellular cues and intracellular signaling. In this work, we applied a PRM-based targeted proteomic method to assess kinome reprogramming during melanoma metastasis, which led to the identification of novel kinases functioning as potential promoters or suppressors for melanoma metastasis. To our best knowledge, this is so far the most comprehensive kinome profiling dataset for melanoma metastasis.

We found that approximately half of the ~300 quantified kinases exhibited differential expression between primary and metastatic melanoma cells (Table S1). In combination with TCGA data, we discovered that JAK3 plays important roles in melanoma progression. Furthermore, the mRNA expression of JAK3 was reduced in the metastatic over primary tumor tissues of melanoma patients, and in metastatic over primary melanoma cell lines (Fig. 4). Therefore, we further investigated JAK3’s roles in melanoma metastasis *in vitro*. Our results showed that reduced JAK3 expression is correlated with increased migration and invasion of cultured melanoma cells (Fig. 5). In addition, the activities of secreted MMPs are negatively correlated with the expression levels of *JAK3* gene in primary and metastatic melanoma cells (Fig. 5), illustrating that JAK3 modulates melanoma cell invasion by regulating the activity of secreted MMPs. Along this line, protein interactome analysis demonstrated that JAK3 interacts with BRMS1, which was shown to suppress melanoma metastasis^38^, and JAK3 co-expresses with another melanoma suppressor, EDAR (Fig. S11)^39^. Therefore, JAK3 may function together with BRMS1 and EDAR to suppress the migration and invasion of melanoma cells.

In summary, our targeted kinome profiling method provides a systematic assessment about kinome reprogramming during melanoma metastasis and our results provides important lines of evidence to support JAK3 as a potential suppressor for melanoma metastasis.

## Experimental Procedures

### Cell culture

IGR-39/IGR-37 (generous gifts from Prof. Peter H. Duesberg, with BRAF^V600E^ and P53^C229fs^)^40^, WM-793/1205Lu (The Wistar Institute, with BRAF^V600E^, P53^WT^ and CDK4^K22Q^) cells were cultured in Dulbecco’s modified eagle medium (DMEM). WM-115/WM-266-4 cells (ATCC, with BRAF^V600D^ and P53^WT^) were cultured in Eagle’s minimum essential medium (EMEM). All culture media were supplemented with 10% fetal bovine serum (Invitrogen, Carlsbad, CA) and penicillin (100 IU/mL). The cells were kept in a humidified atmosphere with 5% CO_2_ at 37 °C. Approximately 2 × 10^7^ cells were harvested, followed by washing with cold PBS for 3 times. The cells were then lysed by incubating on ice for 30 min with CelLytic M cell lysis reagent (Sigma) with 1% protease inhibitor cocktail. After a 30-min centrifugation at 9,000 g and at 4 °C, the resulting supernatants of cell lysates were collected. For SILAC labeling, the cells were cultured in SILAC medium containing unlabeled lysine and arginine, or [^13^C_6_, ^15^N_2_]-lysine and [^13^C_6_]-arginine for at least five cell doublings. The initial passage numbers for the melanoma cells were: WM-115 (p9), WM-266-4 (p6), IGR-39 (p4), IGR-37 (p7), WM-793 (p16), 1205Lu (p70).

### Plasmids and siRNAs

The sequences for siJAK3 were 5′-GGGUCCUUCACCAAGAUUU-3′ and 5′-CCAUGGUGCAGGAAUUUGU-3′ (Dharmacon Inc)^41^, and RNAiMAX (Invitrogen) was employed as the transfection reagent following the supplier’s recommended protocol, where non-targeting siRNA (Dharmacon, D-001210-02-20) was the control. The MIG and the MIG-JAK3 (wild-type) plasmids were kindly provided by Dr. Kara Johnson at Oregon Health and Science University.

### Tryptic digestion of whole-cell protein lysates, and LC-PRM analyses

A previously reported filter-aided sample preparation (FASP) protocol was used to generate tryptic peptides for LC-PRM analysis^12,42^. Four replicates (2 forward and 2 reverse SILAC labeling experiments) of lysates from the WM-115/WM-266-4 pair and 2 replicates (1 forward and 1 reverse SILAC labeling experiments) each of lysates from the IGR-39/IGR-37 and WM-793/1205Lu pairs were prepared for LC-PRM analyses. Peptide mixtures (500 ng) were subsequently dried in a Speed-vac, desalted with OMIX C18 pipette tips (Agilent Technologies), and analyzed by LC-MS and MS/MS on a Q Exactive Plus quadruple-Orbitrap mass spectrometer (Thermo Fisher Scientific) in the PRM mode. The mass spectrometer was coupled with an EASY-nLC 1200 system, and detailed LC-MS/MS conditions were described elsewhere^12^.

All raw files were processed using Skyline (version 3.5) for plotting the extracted-ion chromatograms and for peak integration^43^, where a previously described Skyline PRM kinome library was employed for PRM data acquisition and analysis^12^. Up to four most abundant distinct peptides for each kinase with at most one tryptic mis-cleavage site were included in the library. For peptide identification and quantification, we selected 6 most abundant y ions found in MS/MS obtained from shotgun proteomic analysis, where a ≤20 ppm mass accuracy was imposed in Skyline for fragment ions during peptide identification. We manually inspected all targeted peptides to make sure that the chromatographic profiles for fragment ions derived from the light and heavy forms of the same peptide can be overlaid. We also ensured that the distributions of relative intensities of multiple transitions associated with the same precursor ion are correlated with the theoretical distribution in the kinome MS/MS spectral library entry, where we imposed a dot product (dotp) value^23^ of at least 0.7. The sum of peak areas from all transitions of light or heavy peptides was used for quantification^44–46^, and no other adjustments were made. The relative standard deviations (RSD) for kinase protein quantification were 17.2%, 16.9% and 16.4% for WM-115/WM-266-4, IGR-39/IGR-37 and WM-793/1205Lu pairs of cell lines, respectively (Table S1). Hence, we imposed a 1.5-fold cutoff when considering the expression levels of a kinase to be significantly different in the paired primary/metastatic melanoma cells.

### TCGA, CCLE and GEO data analysis

OncoLnc was employed for the analysis of The Cancer Genome Atlas (TCGA) data to reveal the correlation between patient survival and kinase gene expression^26^, where the top and bottom quartiles of expression values were considered as high and low groups, respectively. The Log-rank p-values for each kinase are calculated individually from the survival distributions of patients with high (top quartile) and low (bottom quartile) expression, and were not adjusted for multiple testing. Survival differences with logrank p-values of <0.05 were considered significant.

Scatter plots were generated for the mRNA expression in melanoma cell lines and tissues based on the data retrieved from the Cancer Cell Line Encyclopedia (CCLE) (https://portals.broadinstitute.org/ccle)^47^, and the GSE8401 dataset from the NCBI Gene Expression Omnibus (GEO). Box-and-whisker plot showing the mRNA levels of JAK3 gene in different types of cancer cells was obtained from CCLE database.

The detailed experimental conditions for Western blot, migration and invasion assay, and gel zymography assay are described in the Supplementary Materials.

## Supplementary information

Supplementary Information.

Table S1.

Table S2.

## Data Availability

All the raw files for LC-PRM analyses of kinases were deposited into PeptideAtlas with the identifier number of PASS01179 (http://www.peptideatlas.org/PASS/PASS01179).

## References

[1] Ubersax JA, Ferrell JE (2007). Mechanisms of specificity in protein phosphorylation. Nat. Rev. Mol. Cell. Biol..

[2] Lemmon MA, Schlessinger J (2010). Cell signaling by receptor tyrosine kinases. Cell.

[3] Blume-Jensen P, Hunter T (2001). Oncogenic kinase signalling. Nature.

[4] Zbytek B (2008). Current concepts of metastasis in melanoma. Expert Rev. Dermatol..

[5] Ascierto PA (2012). The role of BRAF V600 mutation in melanoma. J. Transl. Med..

[6] Rask-Andersen M, Zhang J, Fabbro D, Schioth HB (2014). Advances in kinase targeting: current clinical use and clinical trials. Trends Pharmacol. Sci..

[7] Chapman PB (2011). Improved survival with vemurafenib in melanoma with BRAF V600E mutation. N. Engl. J. Med..

[8] Sharma P, Hu-Lieskovan S, Wargo JA, Ribas A (2017). Primary, adaptive, and acquired resistance to cancer immunotherapy. Cell.

[9] Miao W (2016). A high-throughput targeted proteomic approach for comprehensive profiling of methylglyoxal-induced perturbations of the human kinome. Anal. Chem..

[10] Xiao Y, Guo L, Wang Y (2014). A targeted quantitative proteomics strategy for global kinome profiling of cancer cells and tissues. Mol. Cell. Proteomics.

[11] Patricelli MP (2007). Functional interrogation of the kinome using nucleotide acyl phosphates. Biochem..

[12] Miao W, Guo L, Wang Y (2019). Imatinib-induced changes in protein expression and ATP-binding affinities of kinases in chronic myelocytic leukemia cells. Anal. Chem..

[13] Duncan JS (2012). Dynamic reprogramming of the kinome in response to targeted MEK inhibition in triple-negative breast cancer. Cell.

[14] Stuhlmiller TJ (2015). Inhibition of lapatinib-induced kinome reprogramming in ERBB2-positive breast cancer by targeting BET family bromodomains. Cell Rep..

[15] Urisman A (2017). An optimized chromatographic strategy for multiplexing In parallel reaction monitoring mass spectrometry: insights from quantitation of activated kinases. Mol. Cell. Proteomics.

[16] Miao W, Wang Y (2019). Targeted quantitative kinome analysis identifies PRPS2 as a promoter for colorectal cancer metastasis. J. Proteome Res..

[17] Miao W, Wang Y (2019). Quantitative interrogation of the human kinome perturbed by two BRAF inhibitors. J. Proteome Res..

[18] Miao W, Yuan J, Li L, Wang Y (2019). Parallel-reaction monitoring-based proteome-wide profiling of differential kinase protein expression during prostate cancer metastasis *in vitro*. Anal. Chem..

[19] Ong S-E (2002). Stable isotope labeling by amino acids in cell culture, SILAC, as a simple and accurate approach to expression proteomics. Molecular & Cellular Proteomics.

[20] Westermark B (1986). Human melanoma cell lines of primary and metastatic origin express the genes encoding the chains of platelet-derived growth factor (PDGF) and produce a PDGF-like growth factor. Proc. Natl. Acad. Sci. USA.

[21] Martin J-M, Luis J, Marvaldi J, Pichon J, Pic P (1989). A human melanoma-derived cell line (IGR39) with a very high number of vasoactive-intestinal-peptide (VIP) receptors. Eur. J. Biochem..

[22] Simon H-G (1996). Identification of differentially expressed messenger RNAs in human melanocytes and melanoma cells. Cancer Res..

[23] de Graaf EL, Altelaar AF, van Breukelen B, Mohammed S, Heck AJ (2011). Improving SRM assay development: a global comparison between triple quadrupole, ion trap, and higher energy CID peptide fragmentation spectra. J. Proteome. Res..

[24] Lo JA, Fisher DE (2014). The melanoma revolution: From UV carcinogenesis to a new era in therapeutics. Science.

[25] Huang DW, Sherman BT, Lempicki RA (2008). Bioinformatics enrichment tools: paths toward the comprehensive functional analysis of large gene lists. Nucleic Acids Res..

[26] Anaya J (2016). OncoLnc: linking TCGA survival data to mRNAs, miRNAs, and lncRNAs. PeerJ Comput. Sci..

[27] Darnell J, Kerr I, Stark G (1994). Jak-STAT pathways and transcriptional activation in response to IFNs and other extracellular signaling proteins. Science.

[28] Ye Q, Kantonen S, Gomez-Cambronero J (2013). Serum deprivation confers the MDA-MB-231 breast cancer line with an EGFR/JAK3/PLD2 system that maximizes cancer cell invasion. J. Mol. Biol..

[29] Degryse S (2018). Mutant JAK3 signaling is increased by loss of wild-type JAK3 or by acquisition of secondary JAK3 mutations in T-ALL. Blood.

[30] Kamran MZ, Patil P, Gude RP (2013). Role of STAT3 in cancer metastasis and translational advances. Biomed. Res. Int..

[31] Xu L (2008). Gene expression changes in an animal melanoma model correlate with aggressiveness of human melanoma metastases. Mol. Cancer Res..

[32] Albini A, Benelli R (2007). The chemoinvasion assay: a method to assess tumor and endothelial cell invasion and its modulation. Nature Protocols.

[33] Bauvois B (2012). New facets of matrix metalloproteinases MMP-2 and MMP-9 as cell surface transducers: outside-in signaling and relationship to tumor progression. Biochim. Biophys. Acta.

[34] Vandooren J, Geurts N, Martens E, Van den Steen PE, Opdenakker G (2013). Zymography methods for visualizing hydrolytic enzymes. Nat. Methods.

[35] Yilmaz M, Christofori G (2009). EMT, the cytoskeleton, and cancer cell invasion. Cancer Metastasis Rev..

[36] Mishra J, Das JK, Kumar N (2017). Janus kinase 3 regulates adherens junctions and epithelial mesenchymal transition through β-catenin. J. Biol. Chem..

[37] Vandamme N, Berx G (2014). Melanoma cells revive an embryonic transcriptional network to dictate phenotypic heterogeneity. Front. Oncol..

[38] Shevde LA (2002). Suppression of human melanoma metastasis by the metastasis suppressor gene, BRMS1. Exp. Cell Res..

[39] Vial J (2019). The Ectodysplasin receptor EDAR acts as a tumor suppressor in melanoma by conditionally inducing cell death. Cell Death Differ..

[40] Bloomfield M, Duesberg P (2016). Inherent variability of cancer-specific aneuploidy generates metastases. Mol. Cytogenet..

[41] Gómez-Valadés AG (2012). Specific Jak3 downregulation in lymphocytes impairs γc cytokine signal transduction and alleviates antigen-driven inflammation *In Vivo*. Molecular Therapy. Nucleic Acids.

[42] Wiśniewski JR, Zougman A, Nagaraj N, Mann M (2009). Universal sample preparation method for proteome analysis. Nat. Methods.

[43] MacLean B (2010). Skyline: an open source document editor for creating and analyzing targeted proteomics experiments. Bioinformatics.

[44] Miao W, Li L, Wang Y (2018). A targeted proteomic approach for heat shock proteins reveals DNAJB4 as a suppressor for melanoma metastasis. Anal. Chem..

[45] Miao W, Li L, Wang Y (2018). Identification of helicase proteins as clients for HSP90. Anal. Chem..

[46] Miao W (2019). HSP90 inhibitors stimulate DNAJB4 protein expression through a mechanism involving N6-methyladenosine. Nat. Commun..

[47] Barretina J (2012). The Cancer Cell Line Encyclopedia enables predictive modelling of anticancer drug sensitivity. Nature.

