# Supplementary material for: A Targeted Quantitative Proteomic Method Revealed a Substantial Reprogramming of Kinome during Melanoma Metastasis
Source: Sci Rep. 2020 Feb 12;10:2485. doi: 10.1038/s41598-020-59572-5 (PMC7015909; doi:10.1038/s41598-020-59572-5)

Supporting Information for

"A Targeted Quantitative Proteomic Method Revealed a Substantial Reprogramming of Kinome during Melanoma Metastasis"

Weili Miao[1], Lin Li[1], Xiaochuan Liu[1], Tianyu F. Qi[2], Lei Guo[2], Ming Huang[2], and Yinsheng Wang[1, 2, *]

[1]Department of Chemistry, and [2]Environmental Toxicology Graduate Program,

University of California, Riverside, CA, 92521-0403, USA.

To whom correspondence should be addressed: Tel.: (951)827-2700; Fax: (951)827-4713; E-mail: Yinsheng.Wang@ucr.edu

Supplementary Materials and Methods

Western blot

Cells, while reaching 50%-80% confluency, were lysed in CelLytic M lysis buffer following the aforementioned procedures. The whole cell lysate (10 μg) was denatured by boiling in Laemmli loading buffer and resolved using SDS-PAGE. Subsequently, the proteins were transferred onto a nitrocellulose membrane at 4°C overnight. The resulting membrane was blocked with PBS-T (PBS with 0.1% Tween 20) containing 5% milk (Bio-Rad) at room temperature for 1 h. The membrane was then incubated sequentially with primary antibody at 4°C overnight and with secondary antibody at room temperature for 1 h. After thorough washing with PBS-T, the HRP signals were detected using Pierce ECL Western Blotting Substrate (Thermo).

Antibodies recognizing human AK1 (Santa Cruz Biotechnology, sc-165981, 1:1000 dilution), JAK3 (Thermo Fishier Scientific, AHO1572, 1:1000 dilution), N-cadherin (Thermo Fishier Scientific, 3B9, 1:1000 dilution), PAK (Santa Cruz Biotechnology, sc-166887, 1:5000 dilution), SCYL3 (Santa Cruz Biotechnology, sc-398328, 1:5000 dilution), and STK26 (Abcam, ab52491, 1:20000 dilution) were employed as primary antibodies for Western blot analysis. Horseradish peroxidase-conjugated anti-rabbit IgG and IRDye® 680LT Goat anti-Mouse IgG (H+L) were used as secondary antibodies. Membranes were also probed with anti-actin antibody (Cell Signaling #4967, 1:10000 dilution) to verify equal protein loading.

Migration and invasion assay

Migration and invasion assays were performed with a Matrigel Transwell Chamber (Corning) with 8-μm pore polycarbonate filters.[1] In the migration assay, 3-6 × 10^4 cells were suspended in 100 μl serum-free medium and were added to the upper chambers of the transwell system. Medium

containing 10% FBS was placed in the lower chamber. Non-migrated cells and media in the upper chamber were removed after incubation for the indicated periods of time, and the migrated cells on the bottom surface of the insert membrane were fixed by incubating with 75% methanol at room temperature for 15 min. The cells were then stained with 0.2% crystal violet in 10% ethanol for 15 min and the insert membranes were imaged under a light microscope. Since the same number of cells were suspended in each well, cell migration was represented by the number of migrated cells.

In the invasion assay, the insert membrane was coated with 200-400 μg/ml matrigel in serum-free medium on the upper chamber and the matrigel-containing medium was removed after incubation at 37°C for 2 h. The cells were then dispersed, stained, and counted in a similar way as described above for the migration assay. Cell invasion was calculated by dividing the number of invaded cells over that of the migrated cells.

Gelatin zymography assay

Medium collected from cultured melanoma cells was concentrated and resolved, under non-reducing conditions, using a 10% SDS-PAGE gel (0.1% gelatin). The gel was washed with 2.5% Triton X-100 (Sigma) to remove SDS and to renature MMP-2 and MMP-9 after electrophoresis, and subsequently incubated in the developing buffer (50 mM Tris-HCl, pH 7.5, 1% Triton X-100, 5 mM $CaCl_2$ and 1 μM $ZnCl_2$) for overnight and stained with Coomassie blue. The bands at approximately 63 and 82 kDa were attributed to MMP-2 and MMP-9, respectively.[2] The relative amounts of MMP-2 and MMP-9 were then quantified based on their band intensities using ImageJ.[3]

References:

1. Albini A, Benelli R. The chemoinvasion assay: a method to assess tumor and endothelial cell invasion and its modulation. *Nat. Protoc.* **2**, 504-511 (2007).

2. Tajhya RB, Patel RS, Beeton C. Detection of matrix metalloproteinases by zymography. *Methods Mol. Biol.* **1579**, 231-244 (2017).

3. Vandooren J, Geurts N, Martens E, Van den Steen PE, Opdenakker G. Zymography methods for visualizing hydrolytic enzymes. *Nat. Methods* **10**, 211-220 (2013).

Figure S1. Differential expression of kinase proteins in primary (WM-115) and metastatic (WM-266-4) melanoma cells. The data represent the mean of results obtained from two forward and two reverse SILAC labeling experiments (four biological replicates, see Table S1 for ratios obtained from individual measurements).

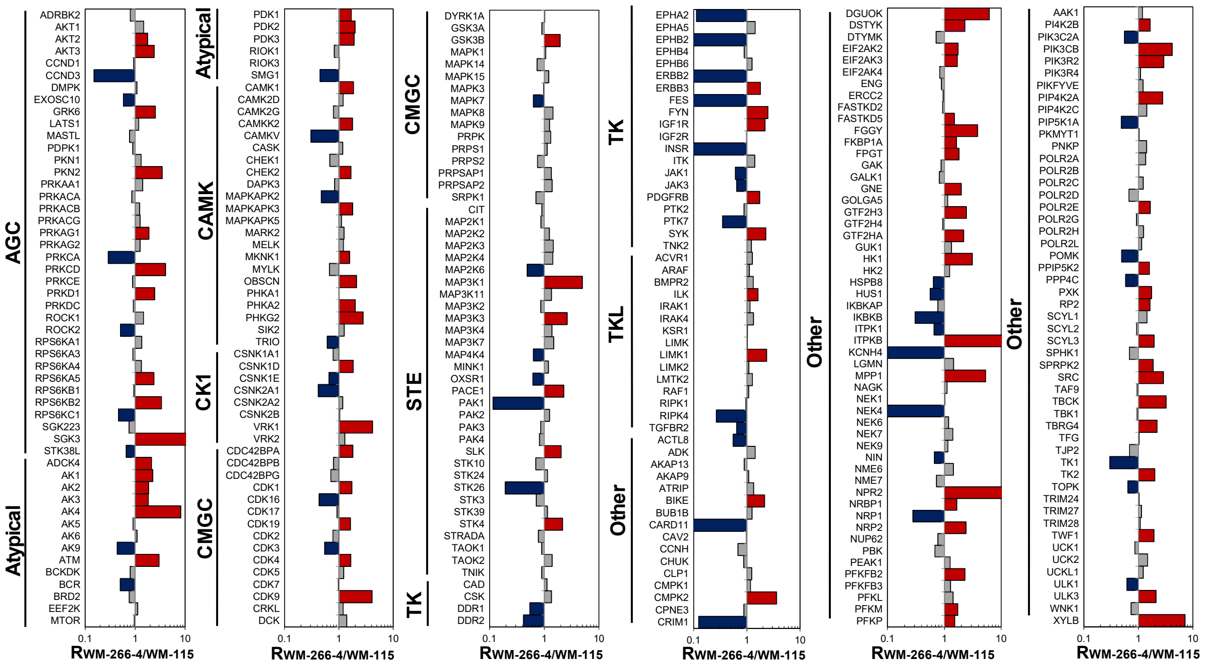

Figure S2. Differential expression of kinase proteins in primary (IGR-39) and metastatic (IGR-37) melanoma cells. The data represent the mean of results obtained from one forward and one reverse SILAC labeling experiments (two biological replicates, see Table S1 for ratios obtained from individual measurements).

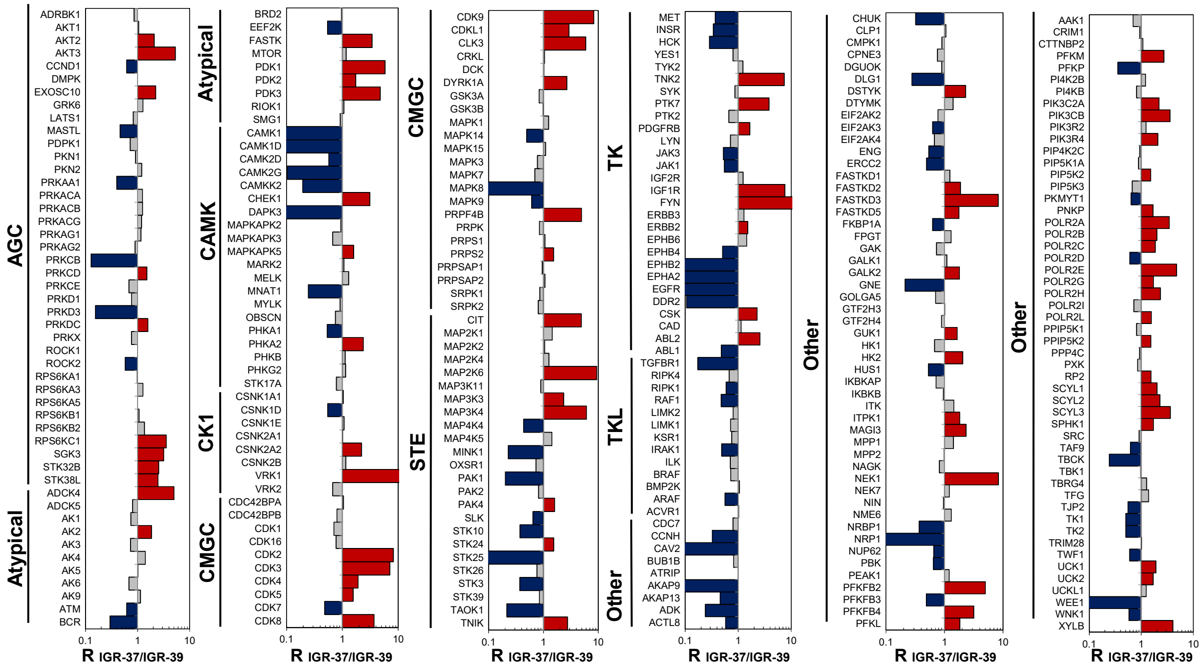

Figure S3. Differential expression of kinase proteins in primary (WM-793) and metastatic (1205Lu) melanoma cells. The data represent the mean of results obtained from one forward and one reverse SILAC labeling experiments (two biological replicates, see Table S1 for ratios obtained from individual measurements).

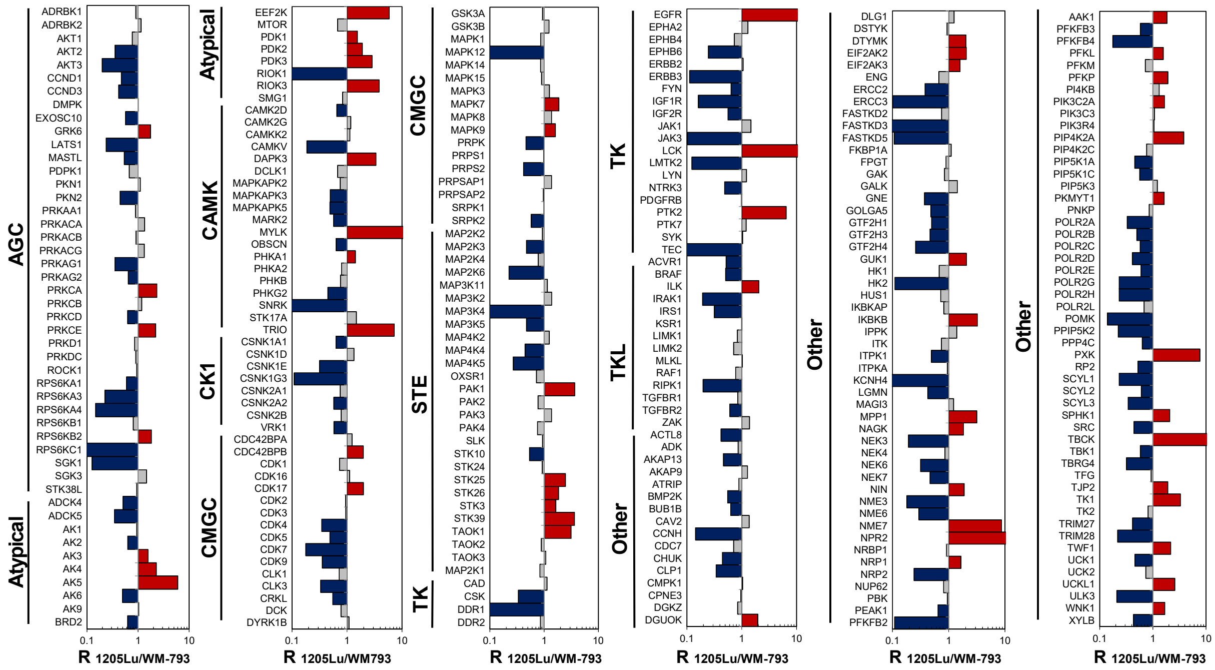

Figure S4. Extracted-ion chromatograms for representative peptides from AK1, JAK3, PAK1, PAK2, SCYL3, and STK26.

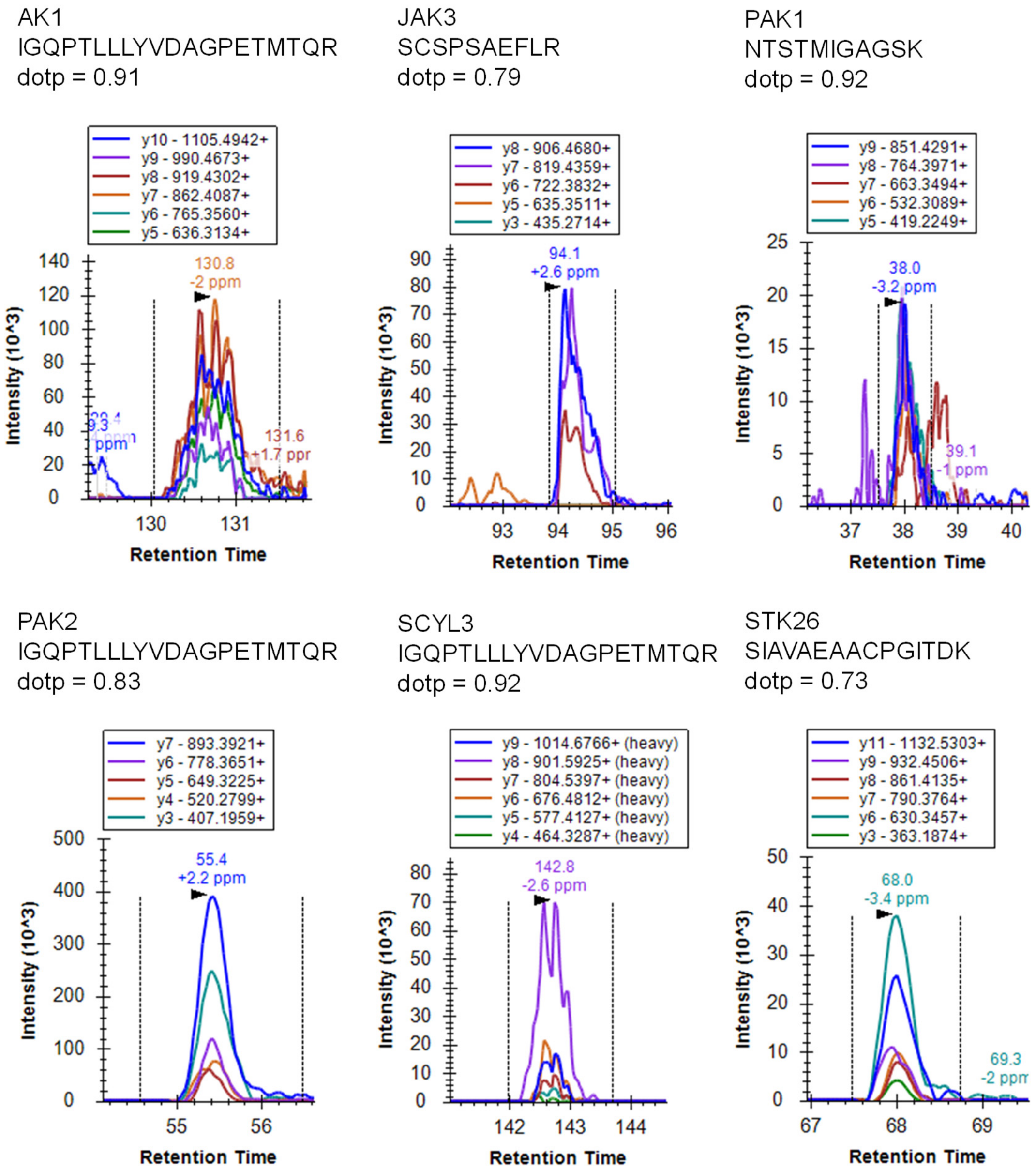

Figure S5. Performance of PRM-based kinome profiling method. (a) Correlation between normalized retention time (iRT) in library and measured RT. (b) PRM traces for a tryptic peptide, IGQPTLLLYVDAGPETMTQR, from AK1 derived from forward (F) and reverse (R) SILAC labeling experiments (y_5, y_6, y_7, y_8, y_9, y_{10} were used for the quantification).

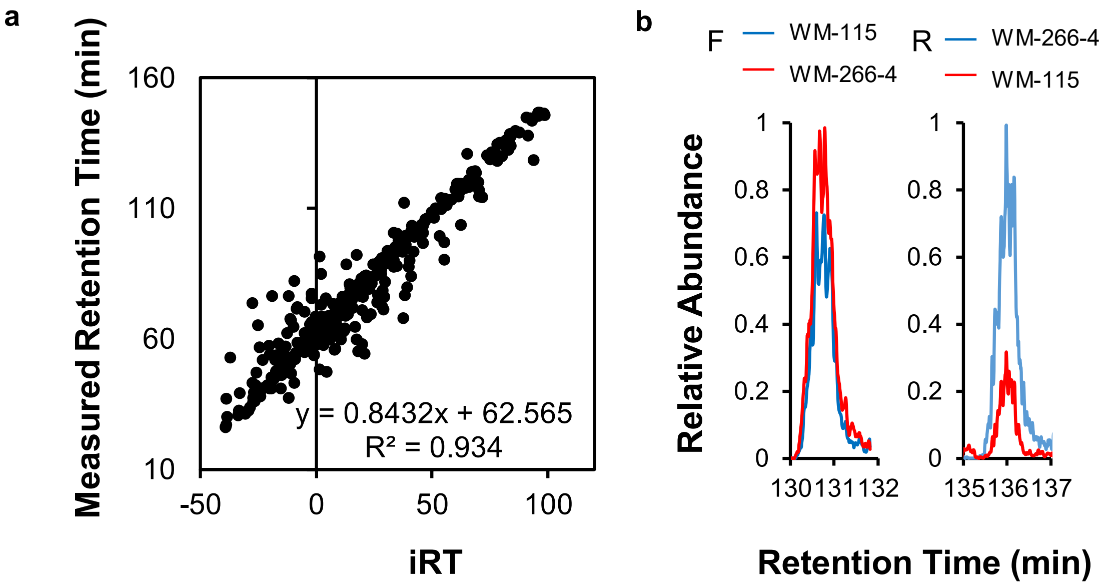

Figure S6. KEGG pathway analysis of the up-regulated kinases in the three lines of metastatic melanoma cells (i.e. WM-266-4, IGR-37 and 1205Lu).

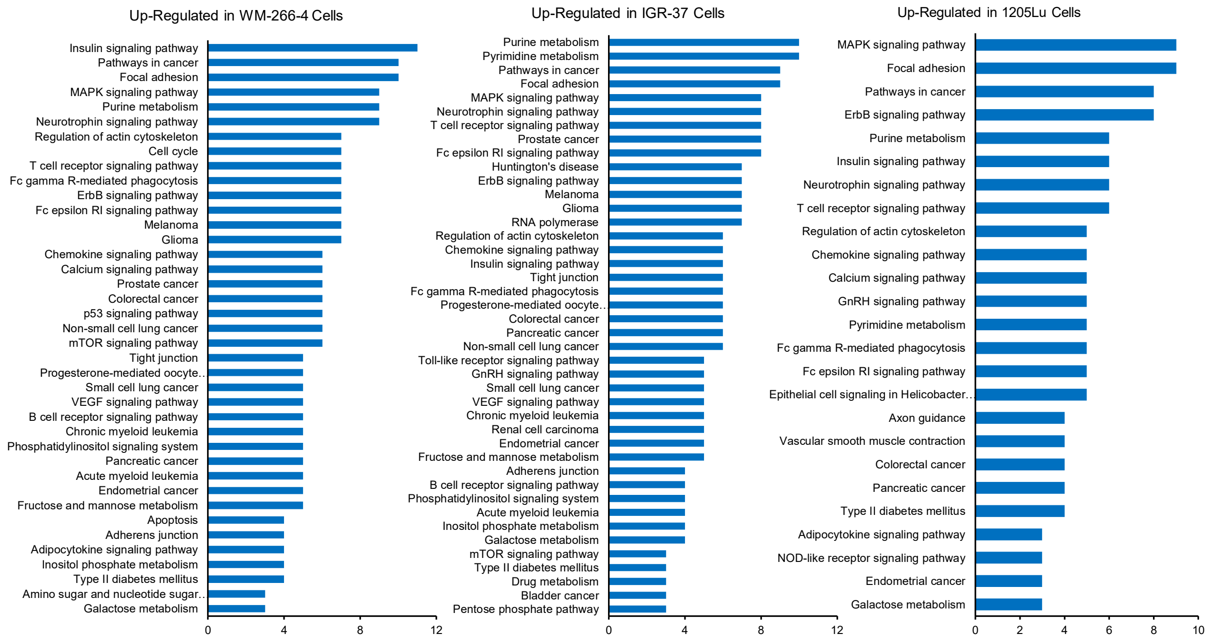

Figure S7. Kaplan-Meier survival plots showing the relationship between the mRNA expression levels of *RPS6KB2, DSTYK, TBRG4, CDK4, POLR2E, FASTKD5, STK26, PAK1, EPHB2* genes and the overall survival of melanoma patients. The data were retrieved from TCGA database and analyzed using OncoLnc (http://www.oncolnc.org/).

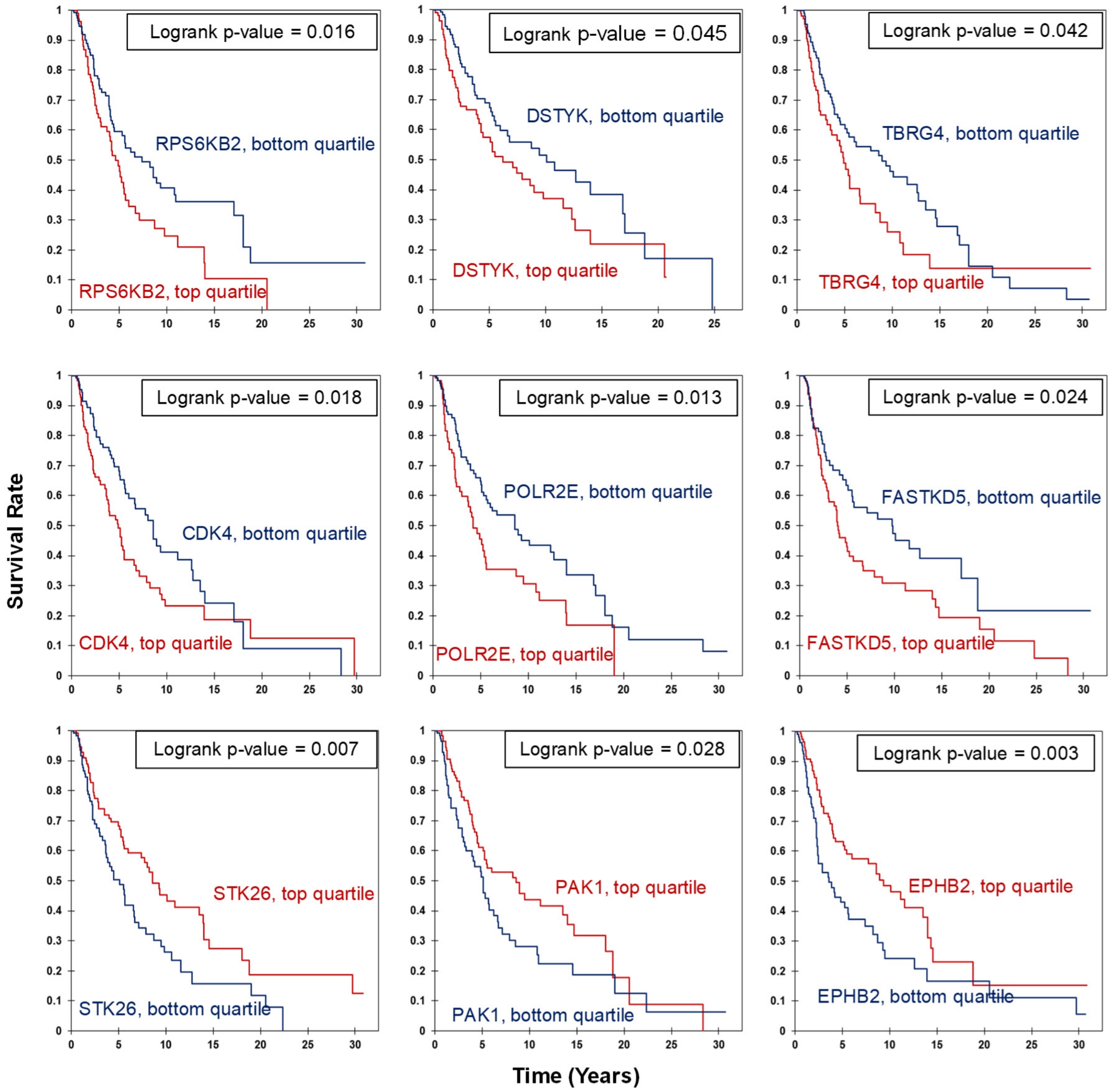

Figure S8. Box-and-whisker plot obtained from CCLE database (https://portals.broadinstitute.org/ccle) showing the the mRNA levels of *JAK3* gene in different types of cancer cells. Box plots depict median (center line), interquartile range (box), smaller of 1.5 times the interquartile range from the box, the minimum–maximum range (whiskers), and outliers (solid circles).

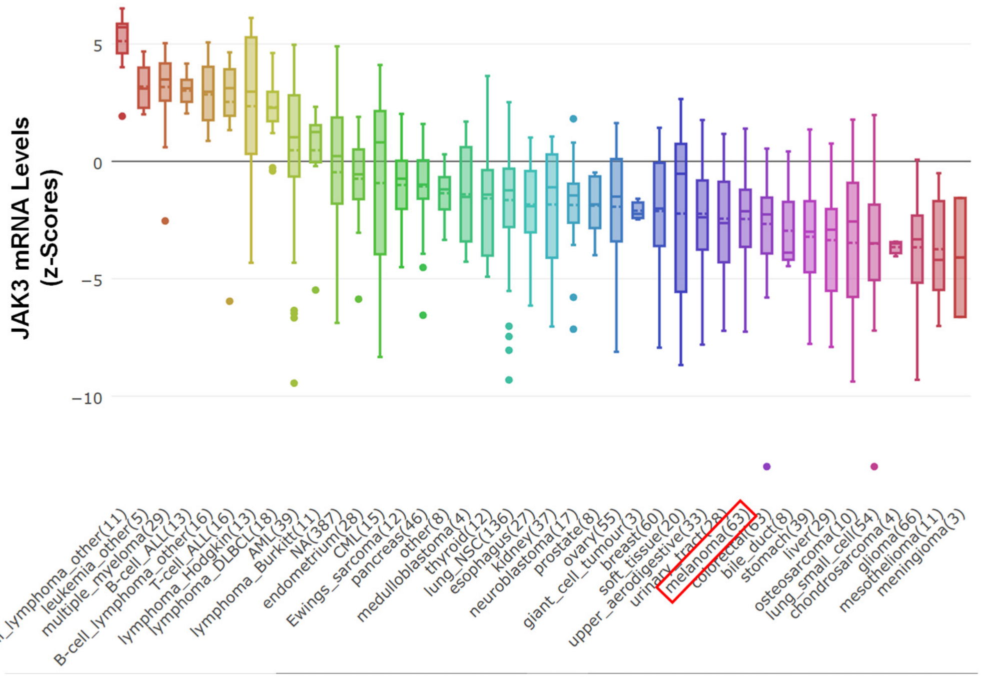

Figure S9. JAK3 modulates the migratory and invasive capacities of cultured melanoma cells through regulating the enzymatic activities of MMP-2 and MMP-9. (a) Western blot results showing the siRNA-mediated knock-down and ectopic expression of JAK3 in WM-115 and WM-266-4 cells, respectively. (b-c) The changes in migratory and invasive abilities (b), and activities of secreted MMP-2 and MMP-9 (c) after ectopic overexpression of *JAK3* gene in WM-266-4 metastatic melanoma cells or siRNA-mediated knock-down of *JAK3* gene in WM-115 cells. The uncropped images for Western blot are provided on page S16.

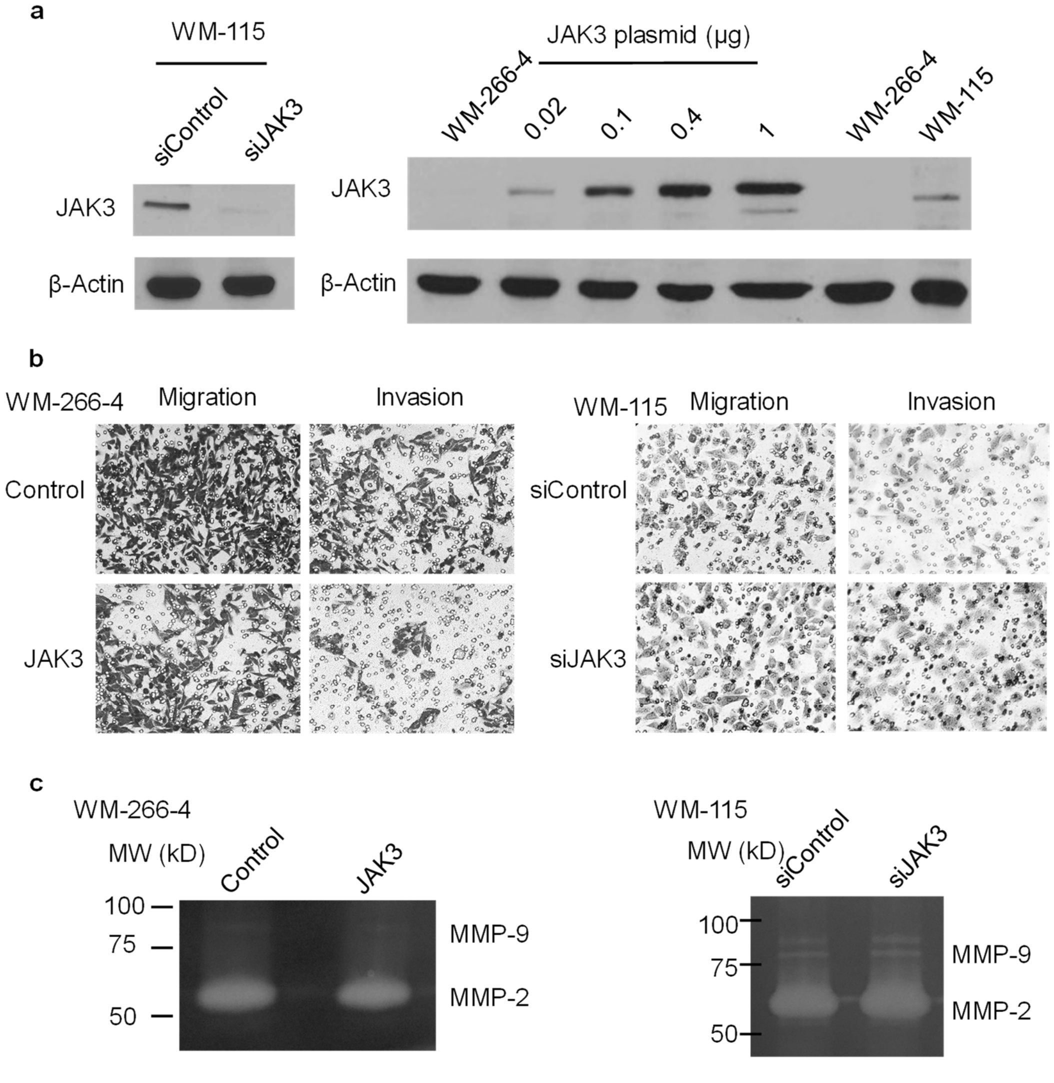

Figure S10. Western blot analysis for the expression of N-cadherin in melanoma cells. (a) Western blot for assessing the relative level of expression of N-cadherin in WM-115 and WM-266-4 cells. (b) Western blot for moitoring the expression levels of N-cadherin protein with or without knock-down of JAK3 in WM-115 cells. (c) Quantitative comparisons of the expression levels of N-cadherin protein in WM-115 cells with or without siRNA-mediated knockdown of JAK3. β actin was employed as the loading control, and the data in (c) represent the mean ± S. D. of the quantification results (n = 3). The uncropped images for Western blot are shown on page S16.

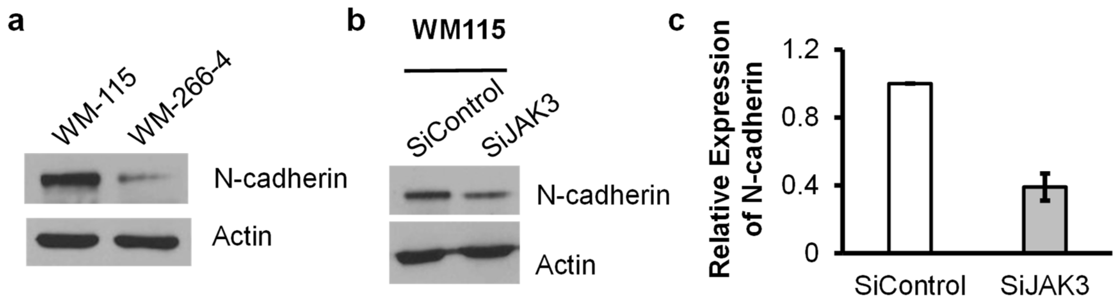

Figure S11. Connections between JAK3 and other melanoma suppressors. (a) Signaling network of JAK3 and BRMS1. The data were analyzed using an online tool (http://genemania.org/). (b) The mRNA expression levels of *JAK3* and *EDAR* genes from 192 melanoma patients. The correlation analysis was performed based on the data were retrieved from TCGA database through cBioPortal (https://www.cbioportal.org/).

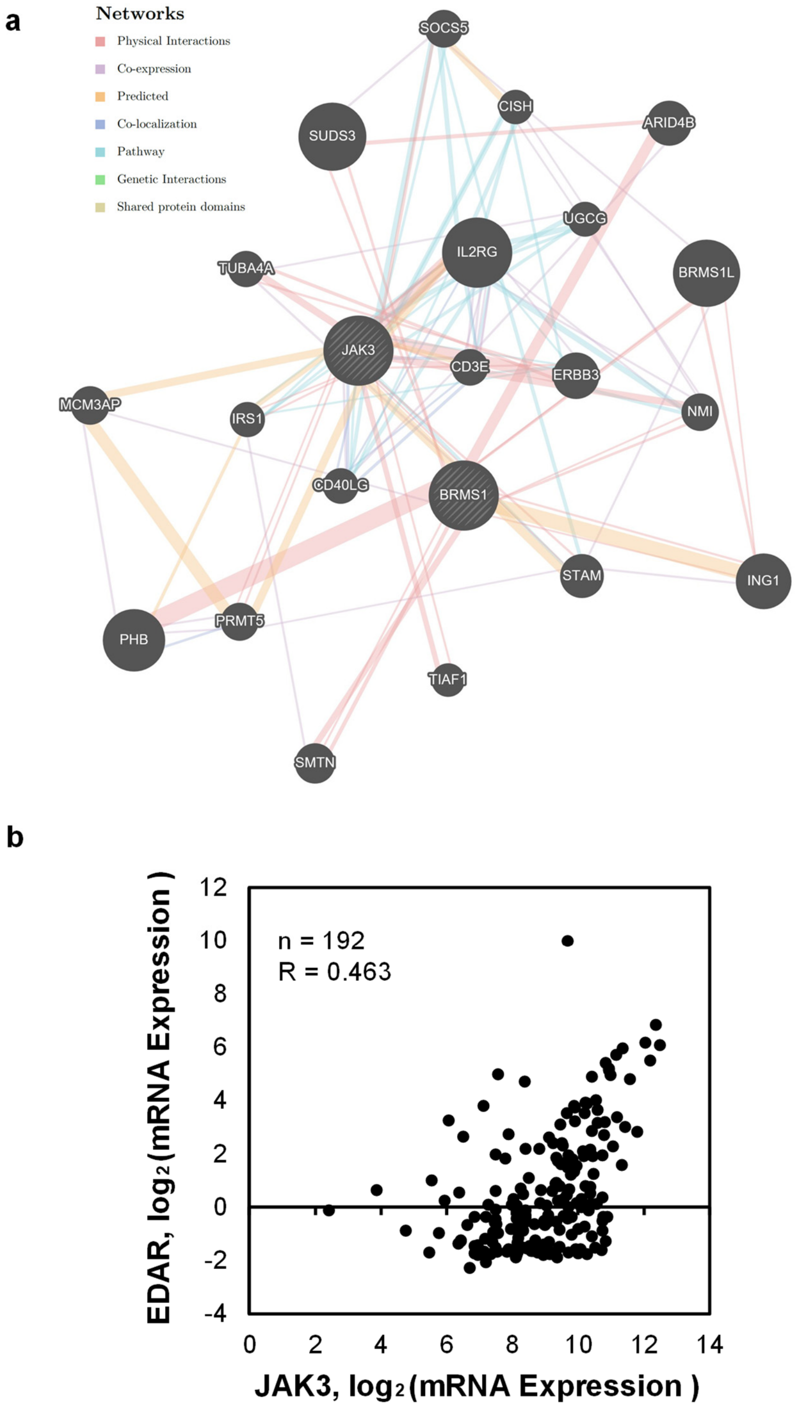

Uncropped WB Images:

Figure 3b

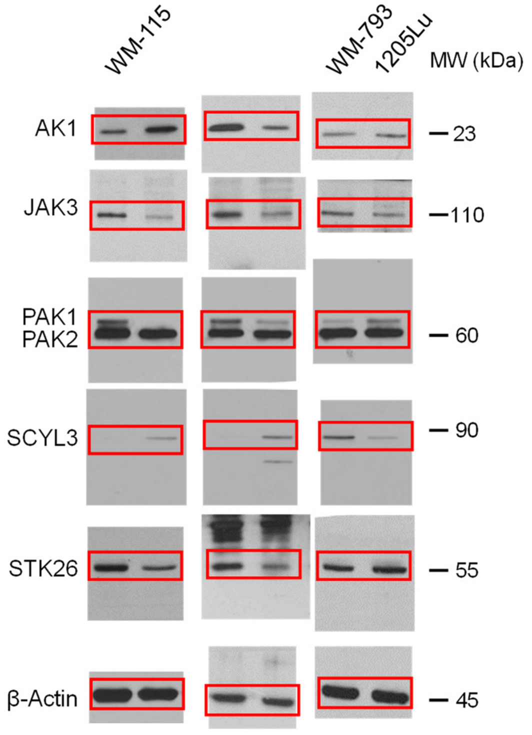

Figure S9a

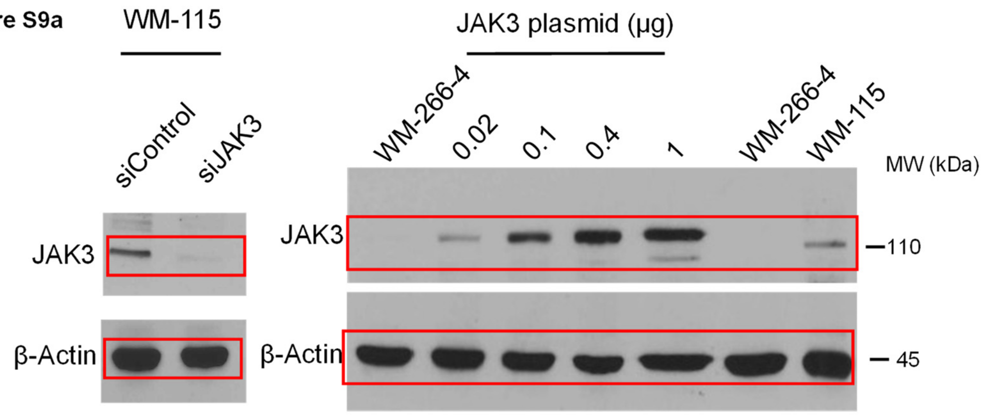

Figure S10a

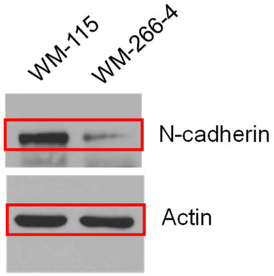

Figure S10b

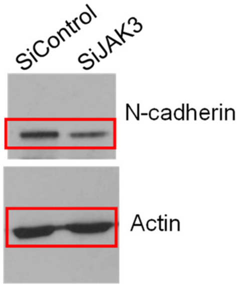

Supplement: Supplementary file 1 — Supplementary Information. [file 41598_2020_59572_MOESM1_ESM.pdf]
